# Predictors of Surgical Outcome After Anterior Cervical Discectomy and Fusion: A Prospective Middle Eastern Cohort Study

**DOI:** 10.7759/cureus.101701

**Published:** 2026-01-16

**Authors:** Ahmed Al-Atraqchi, Rawan Alatraqchi, Yasoob A Alaameri

**Affiliations:** 1 Neurological Surgery, Dr. Saad Al-Witry Neuroscience Hospital, Baghdad, IRQ; 2 Emergency Medicine, Calderdale and Huddersfield Foundation Trust, Halifax, GBR; 3 Neurological Surgery, Baghdad Medical City Complex, Baghdad, IRQ

**Keywords:** acdf outcomes, anterior cervical discectomy and fusion, cervical disc prolapse, neurosurgery, radiculopathy, symptom duration

## Abstract

Background

Cervical disc prolapse is a common cause of radiculopathy and myelopathy. Anterior cervical discectomy and fusion (ACDF) is the standard surgical treatment, but prognostic factors such as age and the duration of symptoms remain debated. This study evaluated outcomes of ACDF and analyzed predictors of recovery.

Methods

Sixty-two patients with cervical disc prolapse underwent ACDF at a single tertiary neurosurgical center. Demographic data, clinical features, operative levels, outcomes, and complications were documented. Outcomes were categorized as improved, unchanged, or deteriorated at final follow-up. Patients were stratified by age (30-39, 40-49, 50-59, and ≥60 years), gender, and symptom duration (<6 months, 6-24 months, and >24 months). Chi-square (χ²) tests were used for associations, with significance at p < 0.05.

Results

The mean age was 49.3 years (range: 31-67); there were 22 men and 40 women. The most common level affected was C5-C6 in 36 (57.1%) patients. Presenting symptoms included upper limb sensory complaints in 54 (87.1%) patients and neck pain in 50 (80.6%) patients. At final follow-up, 52 (83.9%) patients improved, eight (12.9%) remained unchanged, and two (3.2%) deteriorated. The duration of symptoms significantly affected outcomes: all patients treated within six months (22) improved, compared with 26 out of 32 (81.3%) in those with 6-24 months and four out of eight (50%) with >24 months (χ² = 11.18; p = 0.0037). Age was also significant, with four out of eight patients aged ≥60 years showing lower improvement (50%) compared with 24 out of 26 (92.3%) in the 50-59 age group (χ² = 8.31; p = 0.040). Gender did not influence outcome (18 of 22 men {81.8%} versus 34 of 40 women {85%}, p = 0.61). Complications occurred in six of 62 patients (9.7%), including transient dysphagia in four (6.5%) and transient hoarseness in two (3.2%), with no mortality.

Conclusion

ACDF is an effective and safe treatment for cervical disc prolapse, achieving high neurological recovery with a low complication rate. Outcomes are significantly influenced by symptom duration and patient age but not by gender. Early surgical intervention provides the most favorable prognosis, supporting prompt referral and management in suitable candidates.

## Introduction

Cervical disc disease is a common cause of neck pain, radiculopathy, and neurological dysfunction, contributing significantly to global morbidity and disability. The cervical spine, due to its high mobility and complex biomechanics, is particularly prone to degenerative changes. Each intervertebral disc consists of a central nucleus pulposus, which absorbs compressive forces, and a surrounding annulus fibrosus that provides tensile strength [1]. The exact etiology of cervical disc degeneration is multifactorial and not fully understood. Contributing factors include aging, degenerative arthritis, repetitive microtrauma or acute injury, genetic predisposition, and systemic or lifestyle-related conditions. Progressive degeneration may result in osteophyte formation and segmental instability, including spondylolisthesis, which can further compromise neural elements and lead to functional impairment. The degeneration of these structures weakens the disc, leading to the herniation and compression of adjacent neural elements [2].

The most frequently affected levels are C5-C6, followed closely by C6-C7, corresponding to regions of maximal motion and stress [3]. Clinically, cervical disc disease may cause varying degrees of discomfort and neurological impairment. Patients commonly present with axial neck pain or radiculopathy characterized by dermatomal pain, paresthesia, or weakness, while in advanced cases, cervical spondylotic myelopathy may occur, manifested by gait disturbance, sphincter dysfunction, and long-tract signs [4,5]. Symptoms may develop gradually as a result of degenerative changes or acutely following trauma and are often exacerbated by neck movement. While some patients respond to conservative therapy, persistent or progressive neurological deficits due to nerve root or spinal cord compression require surgical intervention, depending on the level and severity of disc pathology.

Magnetic resonance imaging (MRI) remains the diagnostic gold standard, allowing the detailed visualization of disc pathology, nerve root compression, and cord signal changes [6]. Electrodiagnostic studies may assist in confirming the level of neural involvement and chronicity of nerve compression, but their role in predicting postoperative functional outcomes remains inconsistent [7].

Additionally, computed tomography (CT) provides complementary information, particularly in the assessment of dorsal osteophytes and ossification of the posterior longitudinal ligament (OPLL) and for preoperative surgical planning. In selected cases, laboratory investigations may also be performed to exclude infection, autoimmune inflammatory arthritis, or metabolic abnormalities that can mimic or exacerbate degenerative cervical spine pathology [2].

The goals of treatment include pain relief, functional improvement, and the prevention of symptom recurrence and disease progression. Anterior cervical discectomy and fusion (ACDF), first described by Smith and Robinson in 1958, remains the cornerstone of surgical management for cervical disc disease [8]. The procedure entails the anterior removal of the degenerated or herniated disc, followed by interbody fusion using autograft, allograft, or synthetic cages to maintain stability and disc height. Multiple studies have confirmed its safety and efficacy, with consistently high rates of neurological improvement and pain relief.

ACDF is a well-established procedure of choice in patients with preserved or correctable cervical alignment. Both single-level and multilevel disc disease can be effectively managed with ACDF, with reported fusion rates of up to 90%-94% in appropriately selected patients. Furthermore, in selected cases of foraminal stenosis, ACDF combined with uncinate process resection may provide superior neural decompression and improved clinical outcomes when performed concurrently, depending on the level and severity of disc pathology [9-12]. Nonetheless, complications such as transient dysphagia, recurrent laryngeal nerve injury, pseudarthrosis, tracheal or esophageal injury, graft-related problems, and adjacent segment disease remain possible [13].

The prognostic value of demographic and clinical factors such as age and the duration of symptoms following anterior cervical discectomy and fusion remains controversial. While several studies have reported no significant association between patient age and postoperative neurological recovery [14], others have demonstrated poorer outcomes in older patients [15]. Similarly, some authors suggest that prolonged symptom duration adversely affects surgical outcomes, whereas others report comparable recovery irrespective of preoperative symptom chronicity [16].

Several additional factors may influence postoperative prognosis following ACDF, including baseline Neck Disability Index (NDI) scores, the presence of myelopathy, operative duration, and postoperative management strategies such as cervical collar use [5].

Globally, neck pain ranks among the leading causes of disability. In the Middle East and North Africa (MENA) region, approximately 17.4 million individuals were affected in 2019, with an age-standardized prevalence of about 3,067 per 100,000 population [17]. MRI-based studies from neighboring countries reveal a high prevalence of cervical spondylosis, disc bulging, and herniation, particularly among individuals over 50 years of age (80%-90%). These findings highlight the significant burden of cervical spine disorders in the region and the importance of outcome-focused research [18].

Although predictors of postoperative outcome following anterior cervical discectomy and fusion have been described previously, most available evidence is retrospective and derived from Western or East Asian populations. Prospective data from Iraq and the wider Middle East and North Africa (MENA) region remain scarce. Given differences in healthcare infrastructure, referral pathways, and the timing of presentation, the generalizability of existing findings to this setting is uncertain. This prospective study therefore not only evaluates clinical outcomes after ACDF in a tertiary neurosurgical center in Baghdad but also provides a quantitative assessment of the impact of age and symptom duration on recovery, thereby offering region-specific evidence to inform clinical decision-making in similar healthcare environments.

## Materials and methods

Study design and setting

This was a prospective study conducted at the Dr. Saad Al-Witry Neuroscience Hospital in Baghdad, Iraq, between June 2023 and June 2025. The study included patients diagnosed with cervical disc prolapse and treated surgically with anterior cervical discectomy and fusion (ACDF).

Patient population

A total of 62 patients were enrolled, including 22 men and 40 women, with ages ranging from 31 to 67 years. All patients presented with clinical features of cervical disc disease, and the diagnosis was confirmed by plain radiography and magnetic resonance imaging (MRI).

We included adult patients (aged ≥18 years) who underwent single or multilevel ACDF for symptomatic cervical radiculopathy or myelopathy between June 2023 and June 2025. Only patients with preoperative clinical and radiological confirmation of cervical disc pathology were eligible. Patients with prior cervical spine surgery, traumatic cervical injury, active infection, or neoplastic disease were excluded.

All patients underwent an initial period of conservative management, including analgesics, physiotherapy, and activity modification, for a minimum of three months prior to surgery. Patients who failed to improve or experienced persistent symptoms despite conservative treatment were considered for surgical intervention. Patients presenting with progressive neurological deficits or clinical myelopathy were deemed unsuitable for prolonged conservative therapy and proceeded directly to surgical management.

Preoperative assessment

**Figure 1:**
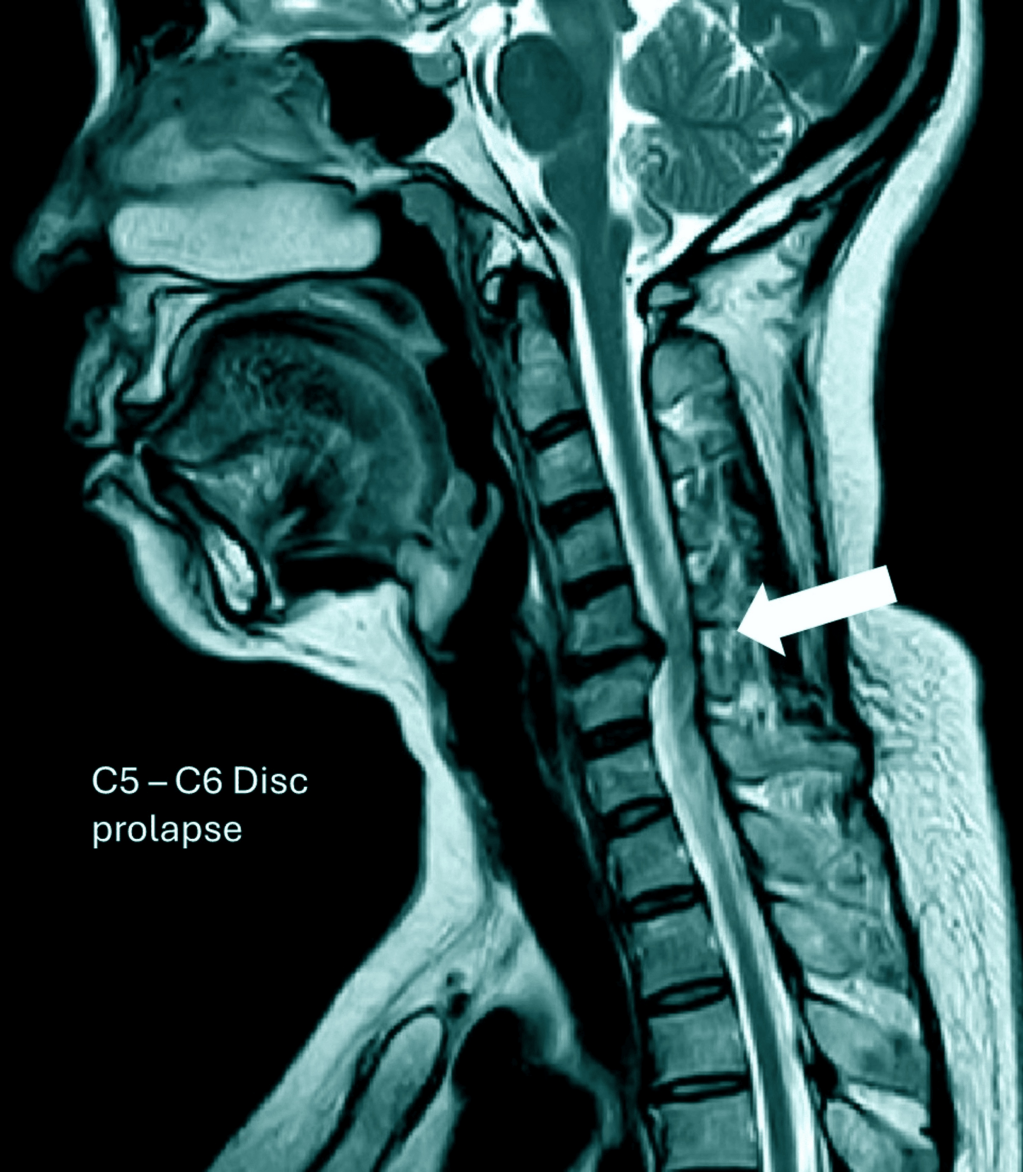
Cervical MRI shows C5-6 intervertebral disc prolapse MRI: magnetic resonance imaging

All patients underwent detailed history taking and neurological examination prior to surgery. Imaging studies included plain radiographs of the cervical spine and MRI for the confirmation of disc pathology and the assessment of neural element compression. See Figure 1.

Surgical technique

All procedures were performed by the same neurosurgical team using a standard ACDF technique. Patients were placed in the supine position under general anesthesia. A transverse right-sided cervical incision was made, and dissection was carried down to expose the cervical spine. The sternocleidomastoid muscle and carotid sheath were retracted laterally, while the trachea and esophagus were retracted medially. The pathological disc and associated posterior osteophytes were removed completely.

**Figure 2:**
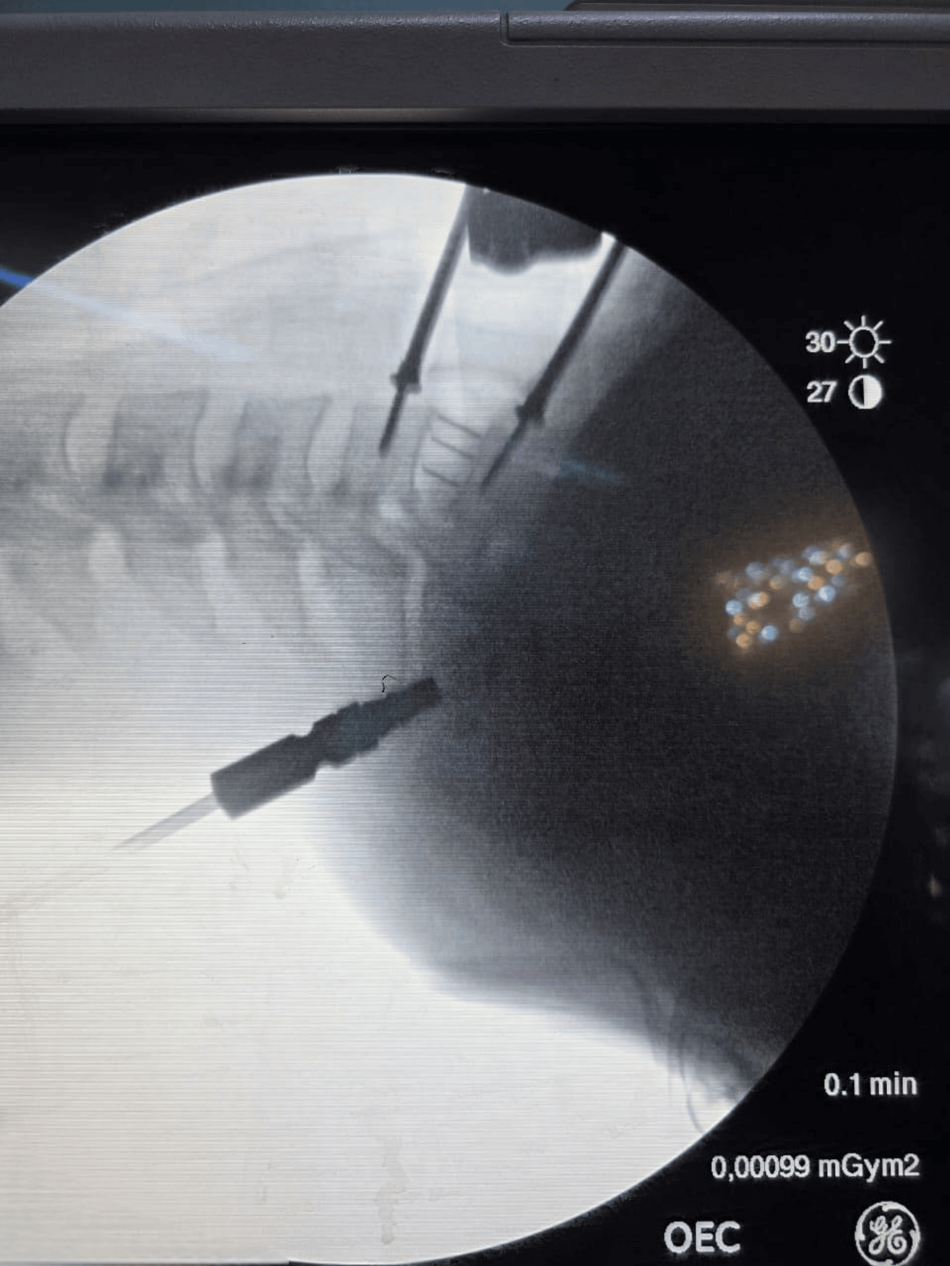
Intraoperative fluoroscopic image of cage insertion

Fusion was achieved using polyetheretherketone (PEEK) cages inserted into the disc space. Intraoperative anteroposterior and lateral fluoroscopy (C-arm) was used to confirm correct level localization and cage placement. Perioperative antibiotic prophylaxis consisted of a single dose of third-generation cephalosporin, repeated postoperatively as required. See Figure 2.

Various surgical strategies for anterior cervical discectomy have been described, including the use of anterior cervical plates, different graft materials, and dynamic or motion-preserving systems such as cervical disc arthroplasty. In the present study, all procedures were performed using standard polyetheretherketone (PEEK) cages without supplemental anterior instrumentation. This approach was adopted to ensure procedural uniformity and because alternative implants, including total disc replacement (TDR) systems and other dynamic devices, were not available at our institution during the study period. As such, the results reflect outcomes achievable with commonly available implants in resource-limited settings.

Postoperative care and follow-up

All patients were fitted with a cervical collar for one month to minimize cervical motion. Neurological evaluation was performed immediately postoperatively and at scheduled follow-up intervals. Follow-up was divided into early follow-up (one month), intermediate follow-up (six months), and late follow-up (12 months). No change in neurological status was noted between six and 12 months.

Postoperative clinical outcomes were categorized as improved, unchanged, or deteriorated based on predefined clinical criteria. Improvement was defined as the partial or complete resolution of preoperative motor or sensory deficits and/or a clinically meaningful reduction in symptom severity compared to baseline. Unchanged indicated no significant difference from the preoperative neurological or symptom status. Deterioration was defined as the worsening of motor or sensory deficits or an increase in symptom severity compared to the preoperative assessment. Outcome evaluation was based on postoperative neurological examination and clinical assessment at follow-up visits. Additionally, radiological evaluation was performed when clinically indicated to assess implant position, alignment, and postoperative changes.

Statistical analysis

Data analysis was performed using IBM SPSS Statistics (IBM Corp., Armonk, NY) for Windows, version 26. Categorical variables were compared using the chi-square (χ²) test. A p-value of <0.05 was considered statistically significant. Proportions are presented with 95% confidence intervals (CI) calculated using the exact binomial (Clopper-Pearson) method in SPSS.

Ethical considerations

Informed written consent was obtained from all patients prior to surgery. The study was approved by the Local Research and Ethics Committee of Dr. Saad Al-Witry Neuroscience Hospital, Baghdad (approval number: LREC/DSWN/2023/005).

## Results

The study included 62 patients with cervical disc prolapse who underwent anterior cervical discectomy and fusion (ACDF). The follow-up duration was 12 months.

Patient demographics

A total of 62 patients were included, with ages ranging from 31 to 67 years. The largest age group was 50-59 years (41.9%), as shown in Table 1.

**Table 1:** Age distribution of the patients

Age groups (years)	n (%)
30-39	12 (19.4)
40-49	16 (25.8)
50-59	26 (41.9)
≥60	8 (12.9)
Total	62 (100.0)

Effect of age on outcome

Improvement was highest in the 50-59-year group (92.3%, 95% CI: 81-100) and lowest in patients aged ≥60 years (50.0%, 95% CI: 15-85). Statistical analysis confirmed a significant association between age and outcome (χ² = 8.31; degrees of freedom {df} = 3; p = 0.040), as shown in Table 2.

**Table 2:** Association between age group and surgical outcome CI: confidence interval

Age groups	n	Improved, n (%) (95% CI)	Unchanged n (%)	Deteriorated n (%)	P-value
30-39	12	10 (83.3) (62-100)	0 (0.0)	2 (16.7)	
40-49	16	14 (87.5) (72-100)	2 (12.5)	0 (0.0)	
50-59	26	24 (92.3) (81-100)	2 (7.7)	0 (0.0)	
≥60	8	4 (50.0) (15-85)	4 (50.0)	0 (0.0)	
Total	62	52 (83.9) (74-94)	8 (12.9)	2 (3.2)	0.04

Gender distribution and outcome

There were 22 men (35.5%) and 40 women (64.5%). Improvement was seen in 81.8% of men (95% CI: 65-98) and 85.0% of women (95% CI: 73-97). The difference was not statistically significant (χ² = 0.26; df = 1; p = 0.61), as demonstrated in Table 3.

**Table 3:** Gender distribution and outcome (chi-square test) CI: confidence interval

Gender	n	Improved, n (%) (95% CI)	Not improved, n (%)	P-value	χ² value
Male	22	18 (81.8) (65-98)	4 (18.2)		
Female	40	34 (85.0) (73-97)	6 (15.0)		
Total	62	52 (83.9) (74-94)	10 (16.1)	0.61	0.26

Level of cervical spine affected

The most common level affected was C5-C6 (57.1%), followed by C6-C7 (23.8%), as demonstrated in Table 4.

**Table 4:** Levels of cervical spine affected *There were 63 affected levels in 62 patients, as one patient had the involvement of two levels

Levels affected	n (%)
C3-C4	2 (3.2)
C4-C5	10 (15.9)
C5-C6*	36 (57.1)
C6-C7*	15 (23.8)
Total levels	63 (100.0)

Clinical features

Upper limb sensory symptoms (87.1%) and neck pain (80.6%) were the most common presenting complaints, as shown in Table 5.

**Table 5:** Clinical features of the patients

Clinical features	n (%)
Upper limb sensory symptoms	54 (87.1)
Neck pain	50 (80.6)
Upper limb weakness	30 (48.4)
Lower limb weakness	22 (35.5)
Gait difficulty	10 (16.1)
Urinary symptoms	8 (12.9)
Lower limb sensory symptoms	2 (3.2)

Outcomes according to the duration of symptoms

The outcomes were analyzed according to the duration of symptoms before surgery and categorized into three groups: <6 months, 6-24 months, and >24 months.

Outcomes showed a clear decline with increasing symptom duration. Improvement was achieved in 100% of patients operated within six months, compared with 81.3% for 6-24 months, and only 50.0% for >24 months. The association between symptom duration and outcome was highly significant (χ² = 11.18; df = 2; p = 0.0037), as demonstrated in Table 6.

**Table 6:** Outcome by the duration of symptoms before ACDF Association between the duration of symptoms and surgical outcome (chi-square test) CI, confidence interval; ACDF, anterior cervical discectomy and fusion

Duration of symptoms	n	Improved, n (%) (95% CI)	Not improved, n (%)	P-value	X^2 ^value
<6 months	22	22 (100.0) (85–100)	0 (0.0)		
6-24 months	32	26 (81.3) (66–96)	6 (18.7)		
>24 months	8	4 (50.0) (15–85)	4 (50.0)		
Total	62	52 (83.9) (74–94)	10 (16.1)	0.0037	11.18

Overall outcomes

At final follow-up, 83.9% of the patients improved (95% CI: 74-94), 12.9% remained unchanged (95% CI: 4-22), and 3.2% deteriorated (95% CI: 0-8), as shown in Table 7.

**Table 7:** Overall outcomes at final follow-up Sensory and motor columns indicate the number of patients with postoperative improvement in sensory symptoms and motor function CI: confidence interval

Outcomes	n (%)	95% CI	Sensory	Motor
Improved	52 (83.9)	74-94	52	20
Unchanged	8 (12.9)	4-22	6	8
Deteriorated	2 (3.2)	0-8	2	2
Total	62 (100)	-		

Complications

Complications were infrequent and transient, with no major morbidity or mortality. The overall complication rate was 9.7% (95% CI: 2-17). Dysphagia occurred in four patients (6.5%, 95% CI: 0-13) and the hoarseness of voice in two patients (3.2%, 95% CI: 0-8), as shown in Table 8.

**Table 8:** Complications after ACDF CI, confidence interval; ACDF, anterior cervical discectomy and fusion

Complications	n (%)	95% CI
Transient dysphagia	4 (6.5)	0-13
Transient hoarseness	2 (3.2)	0-8
Total	6 (9.7)	2-17

## Discussion

In this prospective series of 62 patients with cervical disc prolapse treated with anterior cervical discectomy and fusion (ACDF), 83.9% achieved neurological and functional improvement at final follow-up, while 12.9% remained unchanged, and 3.2% deteriorated. The overall complication rate was low (9.7%) and transient, with no major morbidity or mortality, confirming the safety and efficacy of ACDF in appropriately selected patients.

The duration of preoperative symptoms was the most significant predictor of outcome. All patients who underwent surgery within six months of symptom onset achieved improvement, compared to 81.3% improvement in those treated after 6-24 months and only 50% in patients with symptoms lasting over two years (p = 0.0037). These results agree with Jenkins et al. [19], who demonstrated that earlier surgical intervention correlates with better neurological recovery and lower disability rates, at the three-month (66.1% versus 43.8%, p = 0.039) and six-month (76.8% versus 53.6%, p = 0.030) postoperative period, and with Burneikiene et al [20], who found that that patients treated within six months experienced significantly greater reduction in arm pain compared to those who waited longer than six months (p = 0.04).

Age-related effects on ACDF outcomes remain inconsistent in the literature. In our cohort, older age was associated with a lower improvement rate (p = 0.040). That is consistent with Hao et al., who identified age of ≥55 years as an independent risk factor for suboptimal outcomes (p < 0.05) [21]. However, other studies have reported no clinically meaningful age effect on patient-reported outcomes. For example, Lee et al. found no difference between patients aged 65-74 years and those ≥75 years in visual analog scale (VAS) axial pain (p = 0.448), VAS arm pain (p = 0.357), or NDI (p = 0.913) after ACDF [22]. Likewise, a multicenter analysis by Scerrati et al. reported that age did not significantly influence functional outcome [23].

The higher proportion of unchanged outcomes observed in patients aged ≥60 years, without corresponding postoperative deterioration, may reflect a combination of biological and selection factors. Advanced age is associated with reduced neural plasticity and longer-standing degenerative changes, which may limit the degree of postoperative neurological recovery despite adequate decompression. At the same time, elderly patients selected for surgery are typically carefully screened based on functional status and comorbidity burden, reducing the risk of postoperative neurological worsening. As a result, surgical intervention in this group may stabilize neurological function rather than produce marked improvement, leading to a higher rate of unchanged outcomes without deterioration.

Gender did not influence surgical results (p = 0.61), in agreement with Anderson et al., who reported no significant sex-related difference in neurological recovery or patient-reported outcomes following ACDF [24]. Thus, outcome appears to be governed primarily by anatomical and temporal factors rather than patient sex.

The female predominance observed in this cohort should be interpreted cautiously. Epidemiological studies have reported a higher prevalence of neck pain and degenerative cervical spine conditions among women in various populations [17]. In addition, differences in healthcare-seeking behavior and sociocultural factors may influence presentation rates in regional settings. However, as occupational exposure, lifestyle factors, and sex-specific biological variables were not formally evaluated in this study, no definitive conclusions regarding causation can be drawn.

The improvement rate in this study compares favorably with large international series reporting 75%-90% success after ACDF [25,26]. The incidence of dysphagia (6.5%) and transient hoarseness (3.2%) was low and comparable to that reported by Fountas et al., who observed dysphagia in 9.5% and recurrent laryngeal nerve palsy in 3.1% of cases [13]. The absence of major complications or mortality further demonstrates that ACDF is a safe procedure when performed in experienced centers.

To our knowledge, this is one of the few prospective studies evaluating ACDF outcomes in the Middle East. While most published data originate from North America, Europe, or East Asia, our findings demonstrate that surgical outcomes in this regional setting are comparable to global results despite differences in healthcare systems, referral patterns, and resource availability. The observation that early intervention within six months yields the best recovery has particular relevance in regional practice, where delayed presentation remains common due to limited access to specialized care. These findings underscore the importance of developing timely referral pathways and public health strategies to minimize diagnostic and treatment delays.

The strengths of this study include its prospective design, predefined and clearly described clinical outcome measures, and the systematic evaluation of key prognostic factors. All procedures were performed by the same neurosurgical team using a standardized surgical technique and perioperative protocol, reducing procedural variability. The study employed well-defined inclusion and exclusion criteria, fixed follow-up intervals, and the transparent reporting of complications, enhancing internal validity and reproducibility. In addition, the findings reflect real-world outcomes in a tertiary neurosurgical center within a resource-limited healthcare setting.

However, several limitations should be acknowledged. The modest sample size and single-center design may limit the generalizability of the findings, and the relatively short follow-up period precluded the assessment of long-term fusion success and adjacent segment disease. Outcome assessment was not blinded, which may introduce observer bias, particularly given the partially subjective nature of clinical and neurological evaluation. In addition, outcomes were primarily based on clinical examination and symptom assessment rather than fully standardized patient-reported outcome measures. Finally, multivariable regression analysis was not performed due to sample size constraints, limiting the ability to identify independent predictors of outcome and control for potential confounding factors such as age and the duration of symptoms. Larger, multicenter prospective studies with longer follow-up and multivariable analysis are warranted to further validate these findings.

## Conclusions

This prospective cohort study demonstrates that ACDF is a safe and effective procedure for cervical disc prolapse, associated with high rates of neurological improvement and a low incidence of complications. Symptom duration and patient age are significant determinants of postoperative outcome, underscoring the value of early surgical intervention. Gender was not associated with recovery. These findings support timely surgical management to optimize long-term neurological prognosis. In resource-limited healthcare settings, the results highlight the potential benefit of strengthening early referral pathways and access to specialized neurosurgical care to improve functional outcomes and reduce long-term disability.
